# Incidence and Determinants of Health Care-Associated Blood Stream Infection at a Neonatal Intensive Care Unit in Ujjain, India: A Prospective Cohort Study

**DOI:** 10.3390/diseases6010014

**Published:** 2018-01-30

**Authors:** Mamta Dhaneria, Sachin Jain, Poonam Singh, Aditya Mathur, Cecilia Stålsby Lundborg, Ashish Pathak

**Affiliations:** 1 Department of Paediatrics, Ruxmaniben Deepchand Gardi Medical College, Ujjain 456006, Madhya Pradesh, India; mamtadhaneria@gmail.com (M.D.); drsachinjain2006@yahoo.co.in (S.J.); drpoonamsingh@yahoo.co.in (P.S.); dr.adityamathur121@gmail.com (A.M.); 2 Department of Women and Children’s Health, International Maternal and Child Health (IMCH) Unit, Uppsala University, SE-751 85 Uppsala, Sweden; 3 Global Health—Health Systems and Policy: Medicines, Focusing Antibiotics, Department of Public Health Sciences, Karolinska Institutet, SE-171 77 Stockholm, Sweden; cecilia.stalsby.lundborg@ki.se

**Keywords:** blood stream health care associated infections, neonates, risk factors, antibiotic use, antibiotic resistance, neonatal intensive care unit, India

## Abstract

Very little is known about laboratory-confirmed blood stream infections (LCBIs) in neonatal intensive care units (NICUs) in resource-limited settings. The aim of this cohort study was to determine the incidence, risk factors, and causative agents of LCBIs in a level-2 NICU in India. The diagnosis of LCBIs was established using the Centre for Disease Control, USA criteria. A predesigned questionnaire containing risk factors associated with LCBIs was filled-in. A total of 150 neonates (43% preterm) were included in the study. The overall incidence of LCBIs was 31%. The independent risk factors for LCBIs were: preterm neonates (relative risk (RR) 2.23), duration of NICU stay more than 14 days (RR 1.75), chorioamnionitis in the mother (RR 3.18), premature rupture of membrane in mothers (RR 2.32), neonate born through meconium-stained amniotic fluid (RR 2.32), malpresentation (RR 3.05), endotracheal intubation (RR 3.41), umbilical catheterization (RR 4.18), and ventilator-associated pneumonia (RR 3.17). The initiation of minimal enteral nutrition was protective from LCBIs (RR 0.22). The predominant causative organisms were gram-negative pathogens (58%). The results of the present study can be used to design and implement antibiotic stewardship policy and introduce interventions to reduce LCBIs in resource-limited settings.

## 1. Introduction

Health care-associated infections (HAIs) are a significant public health problem resulting in increased morbidity, mortality and increased hospital stay lengths and health care costs [1]. Neonates admitted to neonatal intensive care units (NICU) are more vulnerable to HAIs because of their underlying susceptibility, to infection and the need for invasive procedures [2].

Various fetal, maternal and NICUs environmental factors contribute toward causing infections in newborns. Apart from the immature immune system, some of the fetal factors that predispose neonates to infections are: low birth weight, gestational age and Apgar score, prolonged hospital stay, invasive procedures, endotracheal tubes, umbilical cauterization, parenteral nutrition, lack of adequate hand washing by hospital personnel and indiscriminate use of antibiotics [1,2,3,4]. Some of the maternal factors that predispose neonates to infections are: the premature rupture of membrane, maternal fever within two weeks prior to delivery, meconium-stained amniotic fluid (MSAF), foul smelling liquor and instrumental delivery, etc. [1,2,3,4]. NICUs environments can be bacteriologically very hostile, containing a wide selection of pathogenic, antibiotic resistant organisms with which the patient becomes colonized [1,2,3,4].

Among the neonatal infections in the NICU, blood stream infections (BSI) are a major cause of concern worldwide [1,4,5,6,7]. In low-middle income countries (LMICs) laboratory confirmed BSI (LCBI) rates are two to three times higher than those of high-income countries [1,3,4,7,8]. In high-income countries, HAI surveillance in the intensive care unit (ICU) plays a major role in infection control as it is essential that HAIs, especially those caused by multi-drug resistant bacteria, be reported [1,4,6]. However, there is a paucity of data from LMICs, including India, where HAIs continue to remain a hidden but serious burden for health systems and patients alike [1,7,9,10,11]. The present study was, therefore, undertaken with an aim to determining the incidence, onset, risk factors, and causative agents associated with blood stream HAIs in a level-2 NICU at C. R. Gardi hospital (CRGH), Ujjain, Madhya Pradesh, India.

## 2. Materials and Methods

### 2.1. Settings

The study was conducted from June 2012 to January 2014 in the eight-bedded NICU, which is a part of the 750-bedded academic hospital CRGH, and which is associated with R. D. Gardi Medical College, Ujjain. The hospital is situated in a semi-urban area approximately six kilometers from Ujjain city in the western part of the province of Madhya Pradesh, India. The hospital caters predominantly to the rural population from the villages surrounding Ujjain city. During the study period, three post-graduate trainees and four consultants provided patient care in NICU. The nurse-to-patient ratio during the study period ranged from 1:8 to 1:12. The bed occupancy remained above 98% during the study period. Our nursery admits a mixed population of low- and high-risk infants. Aseptic non-touch techniques and maximum aseptic precautions were available during the study period, but compliance with these measures was not monitored.

### 2.2. Definitions

In order to define both clinical sepsis and laboratory-confirmed blood stream infections (LCBIs), the standard criteria for HAI surveillance—as defined by the Centers for Disease Control and Prevention’s (CDC’s) National Nosocomial Infection Surveillance System (NNIS) and the National Healthcare Safety Network (NHSN)—were used [12]. Primary BSI was defined as: (1) a patient with a recognized pathogen cultured from one or more blood cultures, where the organism cultured was not related to an infection at another site; or (2) a patient found to have a common skin contaminant (e.g., coagulase-negative staphylococci, viridans group streptococci or micrococci, diphtheroids, *Bacillus* sp., or *Propionibacterium* sp.) cultured from two or more blood cultures [13]. Additionally, the presence of at least one of the signs and symptoms was necessary for the infection diagnosis, including thermal or hemodynamic instability, apnea, milk or glucose intolerance, respiratory distress, hemodynamic instability or underactivity/lethargy [14]. Ventilator Associated Pneumonia (VAP) was defined as per NNIS definitions [12].

LCBIs in neonates admitted in NICU and presenting within the first 72 h of life were defined as early-onset sepsis, while those presenting after 72 h of life were defined as late-onset sepsis [15]. New Ballard scoring was used to calculate the gestational age of newborns after birth [16]. The babies were classified as appropriate, small or large for the gestational age (SGA or LGA) according to Lubchenco and Battaglia charts [17]. The birth weight of the neonates was categorized by adopting the NNIS, USA for high-risk neonatal intensive care units. Three birth weight categories—1001–1500 g, 1501–2500 g, >2500 g—were used [18]. Infants who weighed <2.5 kg at birth were considered low-birth-weight infants, including those small for gestational age, while infants who weighed <1500 g were considered very low-birth-weight infants, and premature infants were those born before 37 weeks of gestation [19]. Mothers with a high risk of chorioamnionitis were defined as having two or more of the following: maternal fever, leukocytosis, maternal tachycardia, uterine tenderness or foul-smelling amniotic fluid [20]. Premature rupture of membranes (PROM) was defined when there was leaking of membranes for more than 24 h before the onset of labor [20]. Minimal enteral nutrition (MEN) in the study was the practice of feeding 10–15 mL/kg/day of enteral feeds.

### 2.3. Inclusion and Exclusion Criteria

Consecutive neonates admitted in NICU during the study period, having a gestational age of more than 28 weeks and diagnosed with LCBIs using the above definitions were included in the study. As there is an inherent uncertainty in ascribing neonatal infection as “maternally acquired” or “hospital acquired” for the study, any infection in the hospital-born neonate was considered a potential HAI. Neonates with a birth weight of less than 1000 g, and neonates transferred to the NICU with a diagnosis of sepsis or showing features of sepsis on admission were not included. LCBIs caused by fungal organisms and anaerobic bacteria were also not included.

### 2.4. HAI Surveillance

A pre-designed questionnaire for the surveillance of LCBIs as HAIs was filled in for all the neonates included in the study. At least two physicians and one of the four resident doctors, who were trained in HAI surveillance, followed all neonates daily until discharge or death.

The questionnaire contained (a) demographic details of the neonates including the name of the neonate and the mother’s name, the age of the baby at the time of admission, the date and time of birth, and the mother’s address; and (b) the maternal, fetal and environmental risk factors associated with LCBIs as HAIs. Appropriate samples for septic screen and blood cultures were collected on all neonates with suspected sepsis. The number of patient-days was defined as the total number of days that patients spent in the NICU during a 28-day period.

### 2.5. Blood Culture Sampling and Processing

Blood cultures were obtained by peripheral puncture. There was no change in the blood culture sampling policy during the study period. Within four hours of receipt, all the samples were plated on blood agar and MacConkey agar medium (HiMedia Laboratories Pvt. Ltd., Mumbai, India). The growth of bacteria within 48 h after blood culture was considered clinically significant. Pathogenic bacteria were identified using standard conventional microbiological methods [21].

### 2.6. Antibiotic Susceptibility Testing

Antibiotic susceptibility testing was performed using the Kirby–Bauer disc diffusion method on Mueller–Hinton agar plates. The disc strengths used were those recommended by the Clinical and Laboratory Standards Institute (CLSI) at the time of the study [22]. CLSI interpretive criteria for susceptibility and resistance were followed [22]. Extended Spectrum Beta-Lactamase (ESBL) were detected phenotypically through a combined disc diffusion method with cefotaxime (30 µg) and cefotaxime/clavulanic acid (30/10 µg), and with ceftazidime (30 µg) and ceftazidime/clavulanic acid (30/10 µg), according to CLSI guidelines [22]; they were confirmed using Vitek 2 [23]. Multidrug-resistant isolates were defined as isolates having co-resistance to at least three antibiotic groups [24]. The ethics committee of RD Gardi Medical College approved the study (approval number 114/2010). The cultures were performed without any cost to the patients, and the results were made available to the concerned physician.

### 2.7. Empiric Antibiotic Therapy in NICU

During the study period, the first-line empiric choice of antibiotic therapy was a third generation cephalosporin (either cefotaxime, ceftriaxone or ceftazidime) with or without an aminoglycoside (either gentamicin or amikacin). The exact choice of antibiotics was made at the discretion of the attending physician. Empirical antibiotic therapy was initiated according to antibiotic guidelines for NICU, if CRP was positive, awaiting culture reports.

### 2.8. Statistical Analysis

The data was entered in EpiData Entry (version 3.1) and then transferred to Stata 12.1 (Stata Corp., College Station, TX, USA) software for statistical analysis. Frequency and percentages are presented for categorical data. A generalized linear regression model (GLM) was used to examine the association of independent risk factors responsible for LCBIs. The independent risk factors were compared between neonates with and without LCBIs (binary outcome variable). The adjusted relative risk (RR) of LCBIs was calculated using multivariate predicted marginal proportions for logistic regression models and included the following independent variables as covariates: sex—male versus female; gestational age—preterm versus term; duration of NICU stay—more than 14 days versus less than 14 days; chorioamnionitis in mother—yes versus no; a history of premature rupture of membranes in the mother—yes versus no; meconium-stained liquor—yes versus no; malpresentation—yes versus no; birth asphyxia—yes versus no; endotracheal intubation—yes versus no; umbilical catheterization—yes versus no; VAP—yes versus no; and minimal enteral nutrition—yes versus no. The means along with the associated 95% confidence intervals (CI) and *p* values were reported from GLMs. A *p* value less than or equal to 0.05 was considered significant.

## 3. Results

A total of 775 neonates were admitted in NICU during the study period and 150 neonates were suspected to have LCBIs. These 150 neonates formed the final cohort. Out of them, 63% (95/150) were male. A total of 65 neonates (43%) were preterm babies according to the New Ballard scoring and the remaining were term babies. A fourth (*n* = 41) of the babies were small for the gestational age (SGA), while the rest were appropriate for the gestational age (AGA); there were no large-for-the-gestational-age babies admitted in the unit during the study period. Out of a total of 150 neonates, a total of 81 neonates were suspected to have early-onset sepsis, while the remaining 69 were suspected to have late-onset sepsis. Fifteen neonates out of 150 (10%) died during the study period. All deaths occurred among preterm neonates, and all had clinical sepsis due to LCBIs.

### 3.1. HAI Incidence

The overall incidence of LCBIs was 31% (i.e., 46/150) (95% CI 23–38%). The incidence of LCBIs among preterm neonates was 45% (i.e., 29/65) (95% CI 32–57%) compared to term neonates, for whom the incidence was 20% (i.e. 17/85) (95% CI 11–29%). The incidence of LCBIs in neonates suspected to have early-onset sepsis (*n* = 81) was 25% (95% CI 15–34%) and that for late-onset sepsis (*n* = 69), was 38% (95% CI 26–49%).

### 3.2. Risk Factors for HAIs

The following risk factors were significantly associated with microbiologically confirmed LCBIs in the multivariate analysis: preterm versus term, NICU stay duration over 14 days versus under 14 days, chorioamnionitis in mother, PROM in mother, neonate born through meconium-stained amniotic fluid (MSAF), malpresentation, endotracheal intubation, umbilical catheterization, and VAP. The initiation of MEN was protective from LCBIs. The details are shown in Table 1. For each increasing day of stay in the NICU the risk for LCBIs increased by 6.6% (SE 2.9%). Additionally, for each incremental day a neonate was on intravenous fluids the risk of LCBIs increased by 12% (95% CI 6–26%; *p* = 0.039).

### 3.3. Antibiotic Use Pattern and Antibiotic Sensitivity Pattern of Pathogenic Isolates

All neonates included in the study were prescribed antibiotics. The mean duration of antibiotics prescribed was 8.2 days. Most (78%) of the patients were prescribed a combination of two antibiotics, while 19% of neonates received a combination of three antibiotics. The most common antibiotic prescribed was amikacin (67%), followed by cefotaxime (30%), ceftriazone (29%), ampicillin (25%), meropenem (24%), vancomycin (20%), ampicillin with cloxacillin (14%), piperacillin with tazobactam (8%), ceftazidime (4%), and metronidazole (3%). The most common combination of antibiotics used was a third-generation cephalosporin with amikacin (59%). Around 20% of the sick neonates were shifted to last resort antibiotics, like meropenem and vancomycin, after initially starting other antibiotics. The antibiotic sensitivity pattern of the isolates is shown in Table 2.

In 58% of cases in our study gram-negative organisms caused blood stream HAIs. *Klebsiella pneumonia* (24%), *Pseudomonas aeruginosa* (13%), *Escherichia coli* (11%), *Staphylococcus aureus* (21%), and Group B Streptococci (17%) were the prominent isolates. Around 73% of *Klebsiella pneumonia* and 80% of *Escherichia coli* were ESBL-producing. The MDR rates were 86%, 82%, 80%, and 92% for *Klebsiella pneumonia*, *Pseudomonas aeruginosa*, *Escherichia coli* and *Proteus* spp., respectively. The MRSA proportion among the *S. aureus* isolates was 35%.

## 4. Discussion

This study, according to our knowledge, is the first study from Central India that reports the incidence and risk factors associated with LCBIs in a level 2 NICU. The results of our study show that several factors are associated with an increased risk of acquisition of LCBIs, including being born preterm, a NICU stay duration of over 14 days, the presence of chorioamnionitis and PROM in the mother, a neonate born through MSAF, malpresentation, endotracheal intubation, umbilical catheterization, and VAP. The initiation of MEN was protective of LCBIs.

The overall incidence of LCBIs in the study was 31%. It is difficult to compare this incidence with other studies without a knowledge of the patient mix admitted by the reporting NICUs and the level of care offered by them. A review of hospital-acquired neonatal infections in developing countries reported that the incidence of culture positivity in South Asia was 15 per 1000 live births [7], which is much lower than that reported by our study. The Delhi Neonatal Infection Study (DeNIS) collaboration study from India, reported a total sepsis incidence of 14% and a culture-positive sepsis incidence of 6·2% (95% CI 5.8–6.6) [9]. However, the DeNIS collaboration study had neonates admitted from level 3 NICUs. Thus, the patient mix of this study cannot be compared with that of our study. The BSI incidence varies from 21% to 39% in nurseries in LMICs, which admit a mixed population of low and high-risk infants [25]. The BSI incidence of 31% reported in our study falls within the range reported in the above study.

A WHO-led review of water, sanitation, and hygiene (WASH) services in 54 LMICs showed severe water shortages in 38% of healthcare facilities, an absence of both water and hand-washing soap in 35% of facilities, and a lack of sanitation in 19% of the facilities [26]. The lack of WASH facilities makes hospitals in LMICs prone to a higher incidence of HAIs [27]. The high rate of neonatal infections in LMICs strongly suggests a lack of adequate hygiene for the peri-partum and neonatal periods in the hospitals [27].

In our study, a neonate born preterm had a RR of 1.75 (95% CI 1.09–2.81; *p* = 0.02) for acquiring an LCBI, compared to a term neonate. It has been shown that, compared to term babies, the incidence of neonatal sepsis in preterm babies is nearly nine times higher, with a birth weight that is between 1000 gm and 1500 gm [4,7]. In our study, chorioamnionitis and PROM increased the risk for LCBIs (RR 3.18 and 2.68, respectively; *p* < 0.001). Chorioamnionitis is very closely related to early-onset sepsis [28]. Chorioamnionitis and PROM in premature babies are associated with higher neonatal mortality, morbidity, and resource use [28]. The risk of early-onset sepsis in a neonate whose mother had a definite chorioamnionitis is approximately 8% [29]. A cohort study reported a 3.5 times increased risk of a sepsis in a premature rupture of membranes in premature neonates [30], compared to our study, which reported a 2.68 times increased risk. In our study, the risk for LCBIs was higher for neonates with MAS (RR 2.32), however there is no evidence that the use of antibiotics for MAS reduces the rate of sepsis in neonates born through meconium-stained amniotic fluid [31]. In our study, fetal malpresentation was associated with LCBIs (RR 3.05, 95% CI 2.05–4.53; *p* < 0.001). Although this was not specifically examined in our study, malpresentation may increase obstetric practices, such as a higher frequency of vaginal examinations, which promote ascending infections in neonates.

Previous studies have shown that a very low birth weight and a longer duration of antibiotic therapy were risk factors for umbilical arterial catheter-related sepsis [32]. A study from China showed that the incidence of umbilical venous catherization related septicemia was at 9.5% [33]. The most common organisms associated with umbilical catheterization related septicemia in our study were *Staphylococcus aureus* and coagulase-negative Staphylococcus, which is similar to what was reported by previous studies [32,33]. Endotracheal intubation has its own complications because of its invasive nature. The risk of infection following endotracheal intubation has not been studied very often in neonates except for VAP. However, there is evidence that endotracheal intubation should be viewed as an important risk for sepsis in neonates [4,7]. It is commonly known that mechanical ventilation is associated with an increased risk of BSI, due to its invasive nature, breech in asepsis during and after the procedure, and during intratracheal suctioning [3,10,34]. The NICU environment per se increases the risk of HAIs, and it is, therefore, no surprise that the NICU stay duration was associated with an increased risk of BSIs. The NICUs are considered to be important focus areas for HAI surveillance and antibiotic stewardship [1,5,6,12]. Minimal enteral nutrition and early total enteral nutrition has been shown to reduce the risk of sepsis and the duration of hospital stays [35].

There is a paucity of data on the bacterial causes of neonatal sepsis in neonates, especially from resource-constrained settings [8]. *Klebsiella species, Escherichia coli, Staphylococcus aureus*, and Group B Streptococci (GBS) predominate in early-onset neonatal sepsis [8]. Conversely, late-onset sepsis is predominantly caused by gram-positive bacteria [8]. Gram-negative rods are responsible for 60–70% of blood-culture-positive infections in the neonatal period [8,34]. In our study, *Klebsiella pneumoniae* was responsible for 22% of the LCBIs. This rate falls within the range of 16–28% reported from different parts of the world [36]. *S. aureus* is responsible for 8–22% of the bloodstream isolates in different regions [9]. Antibiotic resistance is common in the HAI isolates for NICUs. In Indian studies, the most common reported pathogens are gram-negative bacteria, and most are resistant to ampicillin and gentamicin, while some are resistant to cephalosporins [9,10,11]. Antibiotic resistances to common first-line agents render treatments costly and force healthcare workers to increase the spiral of antibiotic use. In our study, we observed considerable variations in the type of antibiotics uses in the NICU. It can be assumed that a considerable proportion of these could be irrational and excessive. However, if the antibiotic-use pattern is seen in light of the resistance pattern of bacterial isolates for HAIs, this use is largely justified.

### Methodological Considerations

Our study has some weaknesses: (1) Hand hygiene is the most important intervention to reduce BSIs in NICUs. We did not measure adherence to hand hygiene as that was not an objective of the study; (2) Despite doing a literature review prior to the start of the study to identify risk factors for BSIs, it is possible that we missed out on some risk factors. Our study has the following strengths: (1) A standard definition for the HAI surveillance was used for the surveillance which is needed for a comparison of results with other international and national studies; (2) Our study assessed the AST pattern for pathogens responsible for HAIs, which generally is lacking in other published studies of HAIs; (3) We have defined the level of care and have reported the LCBI rates according to the term and preterm, which is usually not reported in published studies from LMICs.

## 5. Conclusions

This study determined the incidence, onset, risk factors, and causative agents for LCBIs in resource-constrained settings in India. The results can be used to identify high-risk neonates for LCBIs in other resource-constrained settings. The study highlights the need for strengthening surveillance for LCBIs in resource-limited set-ups. More studies are needed to study the effect of interventions on the modifiable risk factors for LCBIs. The results of the present study will be used to design and implement an antibiotic stewardship policy and introduce interventions to reduce LCBI in our settings.

## Figures and Tables

**Table 1:** Multivariate analysis of neonatal and maternal characteristics associated with laboratory-confirmed blood stream infections in a cohort of 150 neonates.

Risk Factors	Total (%) *	HAI		RR	95% CI	*p* Value
	150	No (%) ^#^	Yes (%) ^#^			
Sex						
Female	55 (37)	40 (73)	15 (27)	Reference	Reference	
Male	95 (63)	64 (67)	31 (33)	1.91	0.71–2.01	0.498
Gestational age						
Term	85 (57)	68 (80)	17 (20)	Reference	Reference	
Preterm	65 (43)	36 (55)	29 (45)	2.23	1.34–3.69	0.002
Duration of NICU stay						
Up to 14 days	115 (77)	85 (74)	30 (26)	Reference	Reference	
>14 days	35 (23)	19 (54)	16 (46)	1.75	1.09–2.81	0.020
Choroamnionitis in mother						
No	142 (95)	103 (73)	39 (27)	Reference	Reference	
Yes	8 (5)	01 (12)	7 (88)	3.18	2.19–4.63	<0.001
PROM in mother						
No	129 (86)	97 (75)	32 (25)	Reference	Reference	
Yes	21 (14)	7 (33)	14 (67)	2.68	1.75–4.11	<0.001
Meconium stained amniotic fluid						
No	134 (89)	98 (73)	36 (27)	Reference	Reference	
Yes	16 (11)	6 (37)	10 (13)	2.32	1.45–3.72	<0.001
Malpresentation						
No	136 (91)	101 (74)	35 (26)	Reference	Reference	
Yes	14 (9)	3 (21)	11 (79)	3.05	2.05–4.53	<0.001
Birth asphyxia						
No	122 (81)	86 (70)	36 (30)	Reference	Reference	
Yes	28 (19)	18 (64)	10 (36)	1.21	0.68–2.13	0.510
ET intubation						
No	128 (85)	99 (77)	29 (23)	Reference	Reference	
Yes	22 (15)	5 (23)	17 (77)	3.41	2.30–5.04	<0.001
Umbilical catheterization						
No	119 (79)	97 (82)	22 (18)	Reference	Reference	
Yes	31 (31)	7 (23)	24 (77)	4.18	2.74–6.38	<0.001
VAP						
No	135 (90)	101 (75)	34 (25)	Reference	Reference	
Yes	15 (10)	3 (20)	12 (80)	3.17	2.16–4.67	<0.001
Minimal enteral nutrition						
No	114 (76)	71 (62)	43 (38)			
Yes	36 (24)	33 (92)	3 (8)	0.22	0.07–0.66	0.008

^#^ Row percentage, * Column percentage PROM-premature rupture of membrane, ET-endotracheal tube, VAP-ventilator associated pneumonia.

**Table 2:** The antibiotic-resistance-pattern of the most prevalent causes of laboratory-confirmed blood stream infections in a cohort of 150 neonates.

Antimicrobial Class/Agent Tested	Activity by Organism (Number Tested)					
	*K. pneumonia* (11)	*P. aeruginosa* (6)	*E. coli* (5)	*Proteus* sp. (4)	*S. aureus* (10)	*CONS* (8)
	R (%)	R (%)	R (%)	R (%)	R (%)	R (%)
Ampicillin	10 (91)	-	4 (80)	3 (75)	8 (80)	7 (88)
Amoxicillin/clavulanate	9 (82)	-	4 (80)	3 (75)	7 (70)	7 (88)
Piperacillin/tazobactam	4 (36)	4 (67)	2 (40)	1 (25)	-	-
Cefuroxime	9 (82)	-	4 (80)	3 (75)	-	-
Ceftriaxone	8 (73)	-	3 (60)	1 (25)	-	-
Cefixime	9 (82)	-	4 (80)	1 (25)	-	-
Ceftazidime	7 (64)	5 (83)	4 (80)	1 (25)	-	-
Ciprofloxacin	9 (82)	5 (83)	3 (60)	3 (75)	-	-
Norfloxacin	9 (82)	-	3 (60)	-	-	-
Ofloxacin	9 (82)	-	3 (60)	2 (50)	-	-
Levofloxacin	-	-	-	-	3 (30)	1 (13)
Gentamicin	7 (64)	4 (67)	3 (60)	1 (25)	-	-
Amikacin	4 (36)	2 (33)	1 (20)	1 (25)	2 (20)	1 (13)
Chloramphenicol	6 (55)	4 (67)	3 (60)	1 (25)	-	-
Tetracycline	10 (91)	5 (83)	4 (80)	2 (50)	-	-
Co-trimoxazole	9 (82)	5 (83)	4 (80)	3 (75)	-	-
Cefoxitin	-	-	-	-	3 (30)	-
Vancomycin	-	-	-	-	0 (0)	0 (0)
Imipenem	-	-	-	-	1 (10)	1 (13)

## References

[1] Allegranzi B., Bagheri Nejad S., Combescure C., Graafmans W., Attar H., Donaldson L., Pittet D. (2011). Burden of endemic health-care-associated infection in developing countries: Systematic review and meta-analysis. Lancet.

[2] Brady M.T. (2005). Health care-associated infections in the neonatal intensive care unit. Am. J. Infect. Control.

[3] Simonsen K.A., Anderson-Berry A.L., Delair S.F., Davies H.D. (2014). Early-onset neonatal sepsis. Clin. Microbiol. Rev..

[4] Vergnano S., Sharland M., Kazembe P., Mwansambo C., Heath P.T. (2005). Neonatal sepsis: An international perspective. Arch. Dis. Child. Fetal Neonatal Ed..

[5] Djordjevic Z.M., Markovic-Denic L., Folic M.M., Igrutinovic Z., Jankovic S.M. (2015). Health care-acquired infections in neonatal intensive care units: Risk factors and etiology. Am. J. Infect. Control.

[6] Folgori L., Bielicki J., Sharland M. (2013). A systematic review of strategies for reporting of neonatal hospital-acquired bloodstream infections. Arch. Dis. Child. Fetal Neonatal Ed..

[7] Zaidi A.K., Huskins W.C., Thaver D., Bhutta Z.A., Abbas Z., Goldmann D.A. (2005). Hospital-acquired neonatal infections in developing countries. Lancet.

[8] Obiero C.W., Seale A.C., Berkley J.A. (2015). Empiric treatment of neonatal sepsis in developing countries. Pediatr. Infect. Dis. J..

[9] Agarwal R., Sankar J. (2016). Characterisation and antimicrobial resistance of sepsis pathogens in neonates born in tertiary care centres in Delhi, India: A cohort study. Lancet Glob. Health.

[10] Nambiar S., Singh N. (2002). Change in epidemiology of health care-associated infections in a neonatal intensive care unit. Pediatr. Infect. Dis. J..

[11] Viswanathan R., Singh A.K., Ghosh C., Dasgupta S., Mukherjee S., Basu S. (2012). Profile of neonatal septicaemia at a district-level sick newborn care unit. J. Health Popul. Nutr..

[12] Horan T.C., Andrus M., Dudeck M.A. (2008). Cdc/nhsn surveillance definition of health care-associated infection and criteria for specific types of infections in the acute care setting. Am. J. Infect. Control.

[13] Hall K.K., Lyman J.A. (2006). Updated review of blood culture contamination. Clin. Microbiol. Rev..

[14] Donahoe M. (2011). Acute respiratory distress syndrome: A clinical review. Pulm. Circ..

[15] Edwards MS B.C., Gershon A.A., Hotez P.J., Katz S.L. (2004). Sepsis in the newborn. Krugman’s Infectious Diseases of Children.

[16] Ballard J.L., Khoury J.C., Wedig K., Wang L., Eilers-Walsman B.L., Lipp R. (1991). New ballard score, expanded to include extremely premature infants. J. Pediatr..

[17] Battaglia F.C., Lubchenco L.O. (1967). A practical classification of newborn infants by weight and gestational age. J. Pediatr..

[18] National Nosocomial Infections Surveillance System (2002). National nosocomial infections surveillance (nnis) system report, data summary from January 1992 to June 2002. Am. J. Infect. Control.

[19] Singh M. (2010). Care of the Newborn.

[20] Tita A.T., Andrews W.W. (2010). Diagnosis and management of clinical chorioamnionitis. Clin. Perinatol..

[21] Murray P.R., Pfaller M.A., Tenover F.C., Yolken R.H. (1999). Manual of Clinical Microbiology.

[22] Clinical and Laboratory Standards Institute (ClSI) (2011). Performance Standard for Antimicrobial Disk Susceptibility Testing.

[23] Winstanley T., Courvalin P. (2011). Expert systems in clinical microbiology. Clin. Microbiol. Rev..

[24] Magiorakos A.P., Srinivasan A., Carey R.B., Carmeli Y., Falagas M.E., Giske C.G., Harbarth S., Hindler J.F., Kahlmeter G., Olsson-Liljequist B. (2012). Multidrug-resistant, extensively drug-resistant and pandrug-resistant bacteria: An international expert proposal for interim standard definitions for acquired resistance. Clin. Microbiol. Infect..

[25] Rosenthal V.D., Lynch P., Jarvis W.R., Khader I.A., Richtmann R., Jaballah N.B., Aygun C., Villamil-Gomez W., Duenas L., Atencio-Espinoza T. (2011). Socioeconomic impact on device-associated infections in limited-resource neonatal intensive care units: Findings of the INICC. Infection.

[26] World Health Organization, UNICEF (2015). Water, Sanitation and Hygiene in Health Care Facilities-Status in Low- and Middle-Income Countries and Way Forward.

[27] Cross S., Afsana K., Banu M., Mavalankar D., Morrison E., Rahman A., Roy T., Saxena D., Vora K., Graham W.J. (2016). Hygiene on maternity units: Lessons from a needs assessment in Bangladesh and India. Glob. Health Action.

[28] Pugni L., Pietrasanta C., Acaia B., Merlo D., Ronchi A., Ossola M.W., Bosari S., Mosca F. (2016). Chorioamnionitis and neonatal outcome in preterm infants: A clinical overview. J. Matern. Fetal. Neonatal Med..

[29] Escobar G.J., Li D.K., Armstrong M.A., Gardner M.N., Folck B.F., Verdi J.E., Xiong B., Bergen R. (2000). Neonatal sepsis workups in infants >/=2000 grams at birth: A population-based study. Pediatrics.

[30] Levine C.D. (1991). Premature rupture of the membranes and sepsis in preterm neonates. Nurs. Res..

[31] Sivanandan S., Agarwal R., Sethi A. (2017). Respiratory distress in term neonates in low-resource settings. Semin. Fetal Neonatal Med..

[32] Landers S., Moise A.A., Fraley J.K., Smith E.O., Baker C.J. (1991). Factors associated with umbilical catheter-related sepsis in neonates. Am. J. Dis. Child..

[33] Hei M.Y., Zhang X.C., Gao X.Y., Zhao L.L., Wu Z.X., Tian L., Tan Y.J. (2012). Catheter-related infection and pathogens of umbilical venous catheterization in a neonatal intensive care unit in china. Am. J. Perinatol..

[34] Khan A.M., Morris S.K., Bhutta Z.A. (2017). Neonatal and perinatal infections. Pediatr. Clin. N. Am..

[35] Nangia S., Bishnoi A., Goel A., Mandal P., Tiwari S., Saili A. (2017). Early total enteral feeding in stable very low birth weight infants: A before and after study. J. Trop. Pediatr..

[36] Pammi M., Zhong D., Johnson Y., Revell P., Versalovic J. (2014). Polymicrobial bloodstream infections in the neonatal intensive care unit are associated with increased mortality: A case-control study. BMC Infect. Dis..

